# Historical view of the effects of radiation on cancer cells

**DOI:** 10.3389/or.2025.1527742

**Published:** 2025-04-30

**Authors:** Saskia Hazout, Christoph Oehler, Daniel R. Zwahlen, Daniel Taussky

**Affiliations:** ^1^ Department of Radiation Oncology, Centre hospitalier de l’Université de Montréal, Montreal, QC, Canada; ^2^ Department of Radiation Oncology, Kantonsspital Winterthur, Winterthur, Switzerland

**Keywords:** radiation, radiobiology, history, double-strand breaks, Microenvironnement

## Abstract

**Introduction:**

Since Röntgen’s discovery of X-rays in 1895, advancements in radiobiology have significantly shaped radiotherapy practices. This historical review traces the evolution of radiobiological theories and their impacts on current therapeutic strategies.

**Materials and Methods:**

Databases such as PubMed were utilized to trace the evolution of concepts in radiobiology.

**Results/Discussion:**

One of the first theories concerning the effect of radiation was Dessauer’s target theory, introduced in the 1920s. He found that damage to critical molecular cellular targets leads to cell death. In the early 20th century, Muller contributed to the understanding of DNA structure and radiation-induced mutations, highlighting theories on the impact of radiation on genetic material and cellular damage. In 1972, Kellerer and Rossi introduced the theory of dual radiation action, which explains that ionizing radiation induces sequential damage to DNA, starting with single-strand breaks and progressing to irreparable double-strand breaks. Recent advances have enhanced the understanding of the effects of radiation on the microenvironment and immune responses, thereby improving therapeutic outcomes. The significance of the sigmoid dose–response curve and the initial shoulder effect were recognized early, leading to theoretical models such as the multitarget single-hit, linear-quadratic and repair-misrepair models. The history of fractionation and the 4R/5R principles have informed today’s ultrahigh fractionation techniques, including single doses of approximately 20 Gy.

**Conclusion:**

Although significant advances have been made toward understanding the effects of radiation on cancerous and healthy tissues, many clinical observations, such as the effects of very high doses or FLASH therapy, remain poorly understood.

## 1 Introduction

Over the centuries, advances in radiobiology have contributed to a deeper understanding of the applications of ionizing radiation and modern therapeutic protocols. In December 1895, Wilhelm Röntgen discovered X-rays at the Würzburg Physics Institute (1). This marked a transformative moment, leading to the invention of the first contact therapy unit. For this discovery, he was awarded the first Nobel Prize in Physics in 1901. Shortly after the discovery, he noted ipsilateral hair loss as well as erythema as a result of X-ray exposure, prompting the exploration of X-rays for treating various lesions (2). During this era, radiation-induced erythema served as a crude dosimetry measure, highlighted by the first reported case of radiation-induced skin cancer by Frieben in 1902, a technician who regularly tested X-rays on his own hand (3). These observations underscore the profound medical implications of X-ray technology. Additionally, Perry Brown, an American radiologist, published his collection of biological essays titled “American Martyrs to Science through Roentgen Rays” in 1936. He died of X-ray-induced cancer in 1950 (4, 5). Early pioneers quickly understood both the advantages and dangers of radiation. This paper provides a summary of the historical development of different theories on the radiobiological action of radiation on cells and its principal protagonists. Advancements and discoveries in radiobiology and quantum biology are traced to gain a better understanding of contemporary radiotherapy practices.

To write a historical review, databases such as PubMed, Google Scholar, and historical scientific books by early pioneers such as Friedrich Dessauer, Nikolay Timofeev-Ressovsky and Douglas E. Lea were consulted. For better comprehension, the review is divided into different sections. First, the discovery of DNA and the effects of radiation on cells and interactions with the microenvironment are discussed, followed by an emphasis on the reasons for the sigmoid shape of the curve, which represents the radiation effect and its evolution over time. The final section focuses on the history of fractionation and the importance of the 4Rs/5Rs in radiotherapy.

## 2 Discovery of DNA and the effects of radiation on cells

In 1869, the Swiss biologist Johann Friedrich Miescher discovered the cell nucleus while conducting experiments on leukocytes. He identified its biochemical differences from other cell organelles because of its resistance to proteases and its high phosphorus content. Miescher hypothesized that this element could play a crucial role in the mechanisms of heredity (6).

In 1902, Theodor Boveri, a German biologist, conducted experiments on sea urchin embryos and demonstrated that deviations from normal chromosomal combinations often lead to cell death, suggesting that cancer may arise from such chromosomal changes (7). As research has progressed, scientists have begun to identify more nuanced mechanisms of cell death. Currently, one distinguishes between direct and indirect cell death. Direct cell death occurs when a cell is directly targeted and killed, while indirect cell death occurs when a cell dies as a result of secondary effects or changes in the cellular environment.

Friedrich Dessauer was a German physicist and a pioneering figure in radiobiology. He revolutionized the application of X-rays through innovative techniques, such as designing a water-cooled Roentgen tube at age 16 in 1897 (8). This early model later served as the foundation for the construction of the first contact therapy unit 20 years later. Research by Dessauer into the interaction between ionizing radiation and living cells continues to influence contemporary therapeutic protocols. Dessauer’s target theory, introduced in the 1920s, posits that cells contain critical molecular targets that, when damaged, lead to cell death (9). According to this theory, the destruction of all targets requires a sufficient radiation dose to induce cell death, highlighting the importance of the dose‒response relationship. These principles are articulated within his “Laws of Homogeneous Irradiation,” which he wrote in 1920 and presented at the 1921 American Roentgen Radiation Society meeting in Washington, DC. These “Laws” were designed to inform and protect against the effects of X-rays (10).

Because of his strong social and political engagement, his strong stance on human rights, scientific freedom, and religious beliefs brought him into opposition to the Nazi regime. He had to leave his position as a professor at the Institute for the Physical Foundations of Medicine at Frankfurt University for Turkey in 1934 (11).

In the early 20th century, American geneticist Hermann Joseph Muller conducted groundbreaking research on DNA, specifically examining the chromosomal structures of *Drosophila* and their implications for heredity. In collaboration with Thomas Hunt Morgan in 1927, Muller devised a method to quantify mutations arising from radiation experiments. His findings unequivocally established that X-rays can induce genetic mutations, a transformative revelation that culminated in his receipt of the Nobel Prize in Physiology and Medicine in 1946 (12, 13). Muller also developed of the concept of the linear no-threshold (LNT) dose response model for hereditary and cancer risk assessment. In 1930, he proposed the existence of the “Proportionality Rule” to describe the dose-response nature of ionizing radiation-induced mutations. The LNT model assumes that even the smallest dose of ionizing radiation carries some risk, with effects accumulating over time. While the Proportionality Rule suggests that the number of mutations caused by radiation is directly proportional to the dose received.

In 1931, Max Delbrück, a young German physicist, redirected his focus toward biophysics and molecular biology. By 1935, he had collaborated with Nikolay Timofeev-Ressovsky and Karl Zimmer on a significant paper proposing an extension of Friedrich Dessauer’s target theory. This extension aimed to estimate gene size on the basis of its sensitivity to ionizing radiation. Referring to the “three-man paper,” they hypothesized that individual quanta of radiation would impact specific “targets” (14). Their research, in which gene stability was measured through mutation rates at varying doses of ionizing radiation and temperatures, suggested the likely nature of a gene as a molecule. Delbrück emigrated from Nazi Germany to the U.S. in 1937 and later received the Nobel Prize in 1969 for a discovery unrelated to radiation effects (9).

In the 1950s, Philip I. Marcus and Theodore T. Puck conducted pioneering studies on the sensitivity of human cells to X-rays in HeLa cells (15). In 1963, Dr. T. Alper proposed that, in addition to DNA, cell membranes could also be targets of radiation, particularly in well-oxygenated cells (16, 17). Furthermore, the indirect effect of radiation plays a significant role in biological damage, often surpassing the direct action in impact. For example, radiation interacting with water molecules in the cell can lead to the formation of free radicals which can trigger chain reactions that amplify biological damage. This hypothesis was supported in the 1970s by studies showing that fat-soluble vitamins and anesthetics could modulate the response to radiation (18). Fat-soluble vitamins may help mitigate radiation-induced damage due to their antioxidant properties. As for anesthetics, they might influence cellular metabolism and oxidative stress pathways, which could indirectly affect how cells respond to radiation exposure.

In 1972, Kellerer and Rossi introduced the theory of dual radiation action (TDAR), emphasizing the role of DNA damage, following Watson and Crick’s discovery of its double-helix structure in 1953 (19). This, after Rosalind Franklin’s X-ray diffraction images of DNA laid the foundation of Watson and Crick’s development of the double helix model (20).

According to TDAR, ionizing radiation induces sequential damage to DNA, beginning as single-strand breaks and progressing to irreparable double-strand breaks and chromosomal aberrations (21, 22).

**FIGURE 1:**
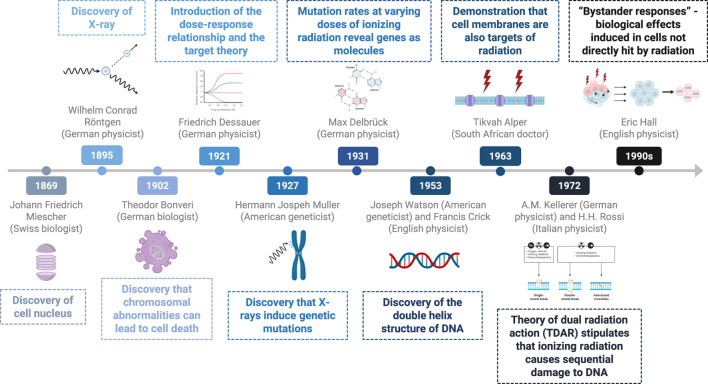
Timeline of key discoveries–effects of radiation on DNA and cells.

Recent research indicates that cell lethality resulting from radiation primarily arises from chromosomal alterations, such as dicentric and acentric fragments, which lead to the loss of genetic material and hinder subsequent cell division, thereby causing mitotic cell death (23). Figure 1 illustrates the history of the discovery of the effect of radiation on DNA.

## 3 History of radiation and cell death

### 3.1 Cell cycle checkpoints

In 1961, Leonard Hayflick and Paul Moorhead discovered the limited lifespan of normal human fetal fibroblasts in culture, a phenomenon termed “cellular senescence,” also known as Hayflick limit. They showed that fibroblast cells could only divide a limited number of times before entering a state of permanent growth arrest, which they termed replicative senescence (24).

Cell cycle checkpoints play critical roles in maintaining DNA integrity postirradiation. These checkpoints serve as protective mechanisms that restrict the transmission of genetic errors, thereby promoting cell survival (25).

In 1961, M. Yamada and T. T. Puck reported that a sublethal dose of X-ray radiation induced a transient G2 premitotic block in HeLa cells (26). It was not until 1968 that a delay in S-phase entry, characteristic of a G1 block, was observed in cultures of normal diploid human cells (27). Furthermore, M. B. Kastan et al. (28) demonstrated in 1992 that primary murine fibroblasts lacking p53 expression lost their G1 checkpoint function following irradiation, whereas those with two wild-type p53 alleles retained this checkpoint.

Additional research in 1996 by Yang Xu and David Baltimore at the Massachusetts Institute of Technology highlighted the critical role of ATM. It activates several key proteins involved in DNA repair, cell cycle checkpoints, and apoptosis, thereby reducing the risk of mutation and cancer development.

Together with the G1 checkpoint, ATM plays a crucial role in the maintenance of cell integrity in response to DNA damage (25, 29).

Recent findings suggest that activation of the G1/S checkpoint occurs gradually over 4–6 h, allowing damaged cells to progress into S phase. In contrast, the G2/M checkpoint is activated more rapidly but requires at least 10 to 20 double-strand breaks (DSBs) for initiation (30–32).

The G0 phase appears after mitosis and serves as a checkpoint, allowing cells to temporarily or permanently exit the cell cycle through postmitotic cell death (33). Studies on yeast fission have shown that cell death can be accompanied by postmitotic arrest, followed by nuclear envelope fragmentation and ultimately plasma membrane permeabilization, leading to postmitotic cell death, as demonstrated in 2008 (34).

Thus, cell irradiation leads to mitotic catastrophe, which results in either senescence (permanent state of proliferation arrest) or cell death, which can occur before, during, or after mitosis (35). Another important finding is the description of post-mitotic cell death and mitotic catastrophe that developed over time. Mitotic catastrophe was first introduced in the scientific literature in the early 1990s and describes a specific type of cell death that occurs during mitosis due to severe mitotic failure. The general term post-mitotic cell death describes irreversible cell death due to DNA damage after mitosis.

### 3.2 From necrosis to apoptosis

In the 1960s, researchers distinguished between two forms of cell death. Classic necrosis is characterized by lysosomal rupture, while the second form involves individual cells transforming into compact cytoplasmic masses containing condensed nuclear chromatin specks with intact lysosomes (36). In 1972, pathologists J.F. Kerr, A.H. Wyllie, and A.R. Currie coined the term “apoptosis” to describe physiological cell death, contrasting it with necrosis. They likened apoptosis to *in vitro* autolysis within phagosomes, devoid of inflammation (37, 38). Apoptosis represents a controlled pathway leading to cellular death, which can also be influenced by mutations in tumor suppressor genes (38).

Necrosis can also arise from the sustained production of growth factors and cytokines, leading to tissue disorganization and dysfunction characterized by fibroblast and vascular proliferation. Clinical manifestations include edema, fibrosis, and telangiectasia (39).

During the 1980s and 1990s, advances in molecular biotechnology shifted apoptosis research from predominantly morphological studies to interdisciplinary fields integral to developmental biology, biogerontology, and cancer research. In 1993, Lowe et al. investigated the proapoptotic role of the p53 gene in the apoptosis of mouse thymocytes and demonstrated that immature p53-mutant thymocytes are resistant to ionizing radiation-induced cell death (40) (Exploring cell apoptosis and senescence to understand and treat cancer 2015). ATM was discovered in 1995 by Yosef Shiloh and highlighted the understanding radiation-induced cell death. ATM mutations are characterized by heightened sensitivity to radiation and a predisposition to cancer. Its involvement in DNA repair pathways underscores its importance in the history of radiation biology and cell death research.

By 1999, preclinical studies underscored the ability of taxanes to increase cancer cell sensitivity to radiotherapy through the phosphorylation of Bcl-2, which induces G2/M arrest and apoptosis via a p53-independent pathway (41). Additionally, experiments on Bax- and Asmase-knockout mice in 2003 revealed that these genes share an antiapoptotic pathway, leading to increased tumor growth and a reduced radiation response due to the increased resistance of endothelial cells to apoptosis (42).

### 3.3 Autophagy

**FIGURE 2:**
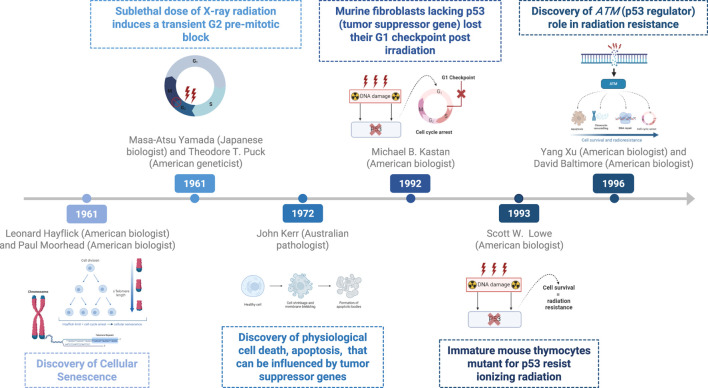
Timeline of key discoveries—radiation and cell death.

Around the turn of the millennium, autophagy, a novel response of cancer cells to radiotherapy was discovered to impact radiation sensitivity (43). However, the precise role of autophagy in radiation treatment remains unclear. Some studies have suggested that increased autophagy may confer cytoprotective effects to cancer cells (44, 45). In the 1960s, Christian de Duve observed that cells encapsulate their contents in membrane-bound vesicles for transport to lysosomes for degradation (46). Yoshinori Ohsumi’s pioneering experiments in the early 1990s using baker’s yeast identified critical autophagy genes and elucidated their mechanisms, demonstrating their similarity to those in human cells (47, 48). In 2016, his Nobel Prize-winning work revealed the mechanisms of autophagy, highlighting its essential role in cellular degradation and recycling (48). Figure 2 delineates the timeline of key discoveries in radiation-induced and cell death.

## 4 History of the interaction between radiotherapy and the microenvironment

Ionizing radiation has been proposed to alter immature tumor blood vessels, potentially leading to disruption of the tumor microenvironment (TME) (49). In 1984, J. Denekamp demonstrated the critical role of the vascular system in tumor survival, highlighting that the absence of collaterals and innervation makes blood vessels attractive targets for ionizing radiation (50). The importance of an intact microvasculature was further underscored in 2003, showing that tumors in apoptosis-resistant mice (asmase- or Bax-deficient) exhibit 200%–400% faster growth than those with a wild-type microvasculature, along with reduced endothelial apoptosis (51).

In 2011, Hallahan and Weinberg described six hallmarks of the TME: “sustaining proliferative signaling, evading growth suppressors, resisting cell death, enabling replicative immortality, inducing angiogenesis, and activating invasion and metastasis” (52). Whereas low doses of ionizing radiation promote angiogenesis via the overexpression of VEGF (53), increasing evidence indicates that high doses of hypofractionated irradiation, commonly used in stereotactic body radiotherapy (SBRT) and stereotactic radiosurgery (SRS), induce secondary cell death through vascular damage occurring days after irradiation, in addition to direct tumor cell death caused by DNA damage (51).

Hypoxia is another factor associated with the TME. Cells that resist direct and indirect cell death mechanisms survive by upregulating antihypoxic signals such as HIF-1α, as demonstrated by Chang W. Song et al. in 2015 (54). Combining radiotherapy with HIF-1α-targeting strategies represents a potential approach to mitigate immunosuppression and enhance therapeutic outcomes (55).

More recently, significant research has focused on targeting the immune system, leading to notable advancements in preventing cancer cells from escaping cell death. Compared with placebo, durvalumab, an anti-programmed death ligand 1 (PD-L1) antibody, significantly prolonged progression-free survival in patients in the phase 3 PACIFIC study published in 2017 (56). Additionally, the efficacy of treatment with nivolumab, a fully human IgG4 monoclonal antibody, in combination with radiotherapy, has been demonstrated through its ability to induce apoptosis by activating PD-1 (57).

There are currently several interesting approaches to exploit the influence of the TME on cancer. Recently, radioligands have been used to target proteins in the tumor microenvironment. Examples include nanobodies against proteins of the extracellular matrix and radioligands to target, for example, chemokine receptors or membrane antigens. This strategy has been effective in reducing immunosuppression and remodeling the TME (58).

Emerging evidence suggests that combining radiotherapy with hyperthermia can increase immune system activity (59). Inflammation may serve as a potential initiator of immune system activation through the release of antigens and cytokines and the promotion of immune cells such as dendritic cells, offering promising advancements in cancer therapy, particularly in the field of immunotherapy (60, 61). Immunologically mediated indirect cell death manifests weeks or months after irradiation following the release of tumor-associated antigens (49).

## 5 Importance of the sigmoid curve of the radiation effect and its evolution

**FIGURE 3:**
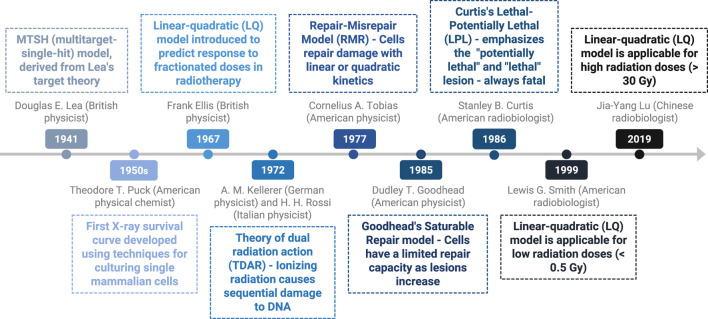
Timeline of key discoveries–importance of the sigmoid curve.

The dose‒response survival curve of cancer cells, which illustrates their response to radiation, is characterized by an initial shoulder, indicating that cell death begins only after a certain radiation dose is delivered. It was recognized early that this shoulder represents the cell’s repair capacity. Over the years, several theoretical models have been developed to explain this phenomenon. Figure 3 delineates the timeline of key discoveries concerning the sigmoid curve. The multitarget-single-hit (MTSH) model, initially derived from Lea’s target theory in 1941, introduces the concept of critical targets within cells that limit cell survival when damaged by radiation. Douglas E. Lea, a British physicist, focused on dose‒response relationships and the consequences of induced cellular damage (62). This model, visualized on a semilogarithmic scale, suggests that reaching a certain damage threshold initiates the linear phase of cell death. This phenomenon was validated in microorganisms exposed to low linear energy transfer (LET) radiation before the discovery of the DNA double helix in 1953 by Watson and Crick (19, 21). The discovery of the double-helix structure of DNA made it possible to better understand the main effect of radiation, the double-strand break. This concept evolved as researchers discovered the effect of radiation on DNA and its repair mechanisms. Advances in molecular biology techniques in the 1970s–1980s provided a better understanding of their mechanisms.

Another significant advancement came from Theodore T. Puck, a physical chemist trained at the University of Chicago under Nobel Prize-winning physicist James Frank. In the 1950s, Puck developed techniques for culturing single mammalian cells and studying their response to various agents, including radiation, using what are now known as HeLa cells (15). Single-cell culture techniques have since been adapted to study the transformation of cells to a neoplastic state. Borek and Sachs (63) demonstrated this transformation via X-rays, whereas Borek and Hall (64) quantified the dose‒response relationship, indicating detectable transformation from 1 rad (0.01 Gy) onward, plateauing at 1% between 100 and 300 rad (1–3 Gy), and declining at higher doses, suggesting increased susceptibility of transformed cells to radiation-induced cell death (65).

The abovementioned two-dose and repair (TDAR) model, proposed by Kellerer and Rossi, explains the shape of the survival curve, particularly the initial shoulder and subsequent linear‒quadratic phases (21, 22). A reinterpretation of TDAR by researchers at the University of Pavia in 2015 introduced the concepts of DNA cluster lesions and lethal chromosomal aberrations, clarifying how these molecular events contribute to the observed linear‒quadratic relationship in the survival curve and further elucidating the impact of radiation on cell survival (20, 65, 66).

Another recent, pivotal theory for understanding radiation effects is the linear‒quadratic (LQ) model, which predicts the response to radiation as a function of the number of fractions and the dose per fraction; this model was pioneered by Frank Ellis. In 1967, Ellis proposed a formula to distinguish the impact of the number of sessions (n) and the total treatment duration (t) on isoeffective dose variations. He later developed the “normal surface dose” (NSD) formula (67). The linear component indicates cellular damage proportional to low doses, suggesting that cells can repair some damage between radiation fractions. Above a certain radiation threshold, a quadratic component appears, indicating that damage increases exponentially with increasing dose, leading to loss of the shoulder and reduced survival. The quadratic portion is also associated with chromosomal aberrations and mutations (23).

The repair-misrepair model (RMR), proposed by Tobias et al. (68), states that cells can repair damage with either linear or quadratic kinetics depending on the extent of damage, with both types of responses contributing to survival curves (69). Goodhead’s saturable repair model (1985) suggests that cells have a limited repair capacity as lesions accumulate, explaining the increased lethality with additional doses and good alignment with the LQ model (70). Finally, Curtis’s lethal-potentially lethal (LPL) model (1986) emphasizes “potentially lethal” and “lethal” lesions to explain differences between the linear and quadratic portions, respectively (21, 71).

In 1999, it was demonstrated that the linear‒quadratic (LQ) model is applicable to low radiation doses (<0.5 Gy) and that the dose‒response curve is similar to that observed for doses ranging from 1 Gy to 6 Gy, which are used for predictive purposes. It is possible that cells do not undergo checkpoint arrest and continue their cell cycle with small DNA damage until tumor suppressor genes, such as P53, act to eliminate damaged cells. However, increased radioresistance was observed at higher doses. This phenomenon may be attributed to the fact that cells are unsynchronized and are in different phases of the cell cycle at the time of irradiation, which could explain the observed hypersensitivity at low doses followed by increased radioresistance, (72, 73).

In 2019, Lu et al. demonstrated that the LQ model is applicable even at high fractional doses of up to 30 Gy often used for non-small cell lung cancer (74). This showed that the LQ model is valid for larger doses per fraction utilized today for SBRT.

Some studies have also invoked the “bystander effect” to explain how unexposed cells respond to radiation through biological signals transmitted by exposed cells, leading to hypersensitivity. This phenomenon was first demonstrated by Nagasawa and Little in 1992, in a model in which only 1% of the studied cells had been irradiated by alpha particles, yet 30% exhibited chromosomal damage, characterized by sister chromatid exchanges (75). Introduced by Eric Hall in 2003, the bystander response describes biological effects induced in cells not directly irradiated. Research distinguishes between experiments in which medium is transferred from irradiated to unirradiated cells, showing effects such as cell death and chromosomal abnormalities, and microbeam studies, which demonstrate effects through gap junctions between neighboring cells. The effect varies with cell type and radiation type and is more pronounced with densely ionizing radiation, such as alpha particles (67). However, studies on clonogenic cell lines have shown an inverse relationship between the “bystander” effect and radiosensitivity/radioresistance, in which cell lines with broader model shoulder regions exhibit greater initial response followed by increased saturation of the mechanism. At higher doses, the signaling mechanisms may already be saturated, and the cells initiating the bystander effect may be unable to produce cytokines, such as tumor necrosis factor (TNF) or interleukin 8, or reactive oxygen species, which would initiate the destruction of surrounding cells (76).

## 6 History of fractionation and the importance of the 4Rs in radiotherapy

### 6.1 History of fractionation

By 1926, dose tolerance estimates were first documented and frequently standardized on the basis of the erythema dose (3). In 1928, a dose‒response curve for skin erythema was subsequently developed (77). Furthermore, Holthusen conceptualized the trade-off between the tumor control probability (TCP) and the normal tissue complication probability (NTCP) in 1936 (78). In 1951, Leksell authored a seminal paper on radiosurgery, chronicling the initial cases treated via X-rays, marking the advent of widespread adoption of the method (79). Since the 1990ies, DJ Brenner and EJ Hall have published alone or together several important studies. Their contributions to the understanding of the biological effects of ionizing radiation and mathematical modeling of radiation effects are seminal. They developed mathematical models to describe the effects of radiation on cells and tissues, helping to predict and understand the outcomes of radiation exposure and investigate cancer risk from radiation (80, 81).

To refine normal tissue dose‒volume tolerance, Rubin (1960s) and Emami et al. (82) developed the Quantitative Analyses of Normal Tissue Effects in the Clinic (QUANTEC) guidelines in the 2000s. These guidelines specify the dose to healthy tissue as a percentage of volume for conventionally fractionated radiation therapy, relying on expert consensus and published data (49, 79).

Conventionally fractionated radiotherapy doses of 1.5–2.0 Gy per fraction are utilized to minimize damage to healthy tissues while effectively treating cancer. In 2013, Cosset et al. traced the historical evolution of early treatment paradigms and discussed optimal fractionation schedules while considering temporal factors (83). Historically, debates within the Curie Foundation have highlighted a compromise between the efficacy of cancer cell killing and minimizing damage to healthy tissue. Régaud, a radiobiologist, emphasized the importance of not delivering too low a dose per fraction over a prolonged period to maximize efficacy. Coutard, a clinician, expressed concern regarding the observed damage to connective and vascular tissue associated with excessively high fractional doses (84).

Hyperfractionation (multiple treatments per day) aims to spare late-responding tissues by reducing the dose per fraction while maintaining effective tumor control (85). In 2004, Harney et al. reported that treating metastatic skin tumors with 0.5 Gy per fraction could enhance cancer control through low-dose fractionation strategies (86). Conversely, hypofractionation, which involves doses higher than 1.8–2 Gy per fraction, delivers larger doses per session but may not offer clear advantages over standard fractionation. Its goal is to balance tumor control by minimizing damage to normal tissues through shorter treatment times using advanced targeting techniques (87). The evidence supports the effectiveness of moderate hypofractionation of 2.25–3.5 Gy (88). More recently, even higher-dose-per-fraction regimens, known as stereotactic radiosurgery (SRS), stereotactic body radiation therapy (SBRT), and stereotactic ablative body radiotherapy (SABR), have been safely implemented. Detailed information about patient responses is provided by sources such as the American Association of Physicists in Medicine (AAPM) Task Group Report TG101 and subsequent updates (52, 84).

The HyTEC (Hypofractionated Treatment Effects in the Clinic) initiative aims to increase estimates of normal tissue complication probability (NTCP) and tumor control probability (TCP) for SRS/SBRT by systematically reviewing and pooling peer-reviewed clinical data, thereby improving clinical practice standards. By mutual agreement between the American Association of Physicists in Medicine (AAPM) and the American Society for Radiation Oncology, HyTEC reports have been featured in the International Journal of Radiation Oncology, Biology, and Physics (89).

With the further development of hypofractionation in the 2010s, research regarding ultrahigh dose rates (FLASH) expanded from *in vitro* studies to investigating their effects on normal tissues *in vivo*.

FLASH radiotherapy (FLASH-RT) leverages ultra-high dose rates to minimize normal tissue damage while maintaining or enhancing tumor control. This phenomenon, known as the FLASH effect, is thought to arise because normal cells have a limited time to initiate harmful biological responses to radiation, whereas cancer cells, due to their altered repair mechanisms, remain susceptible to radiation-induced damage (90).

**FIGURE 4:**
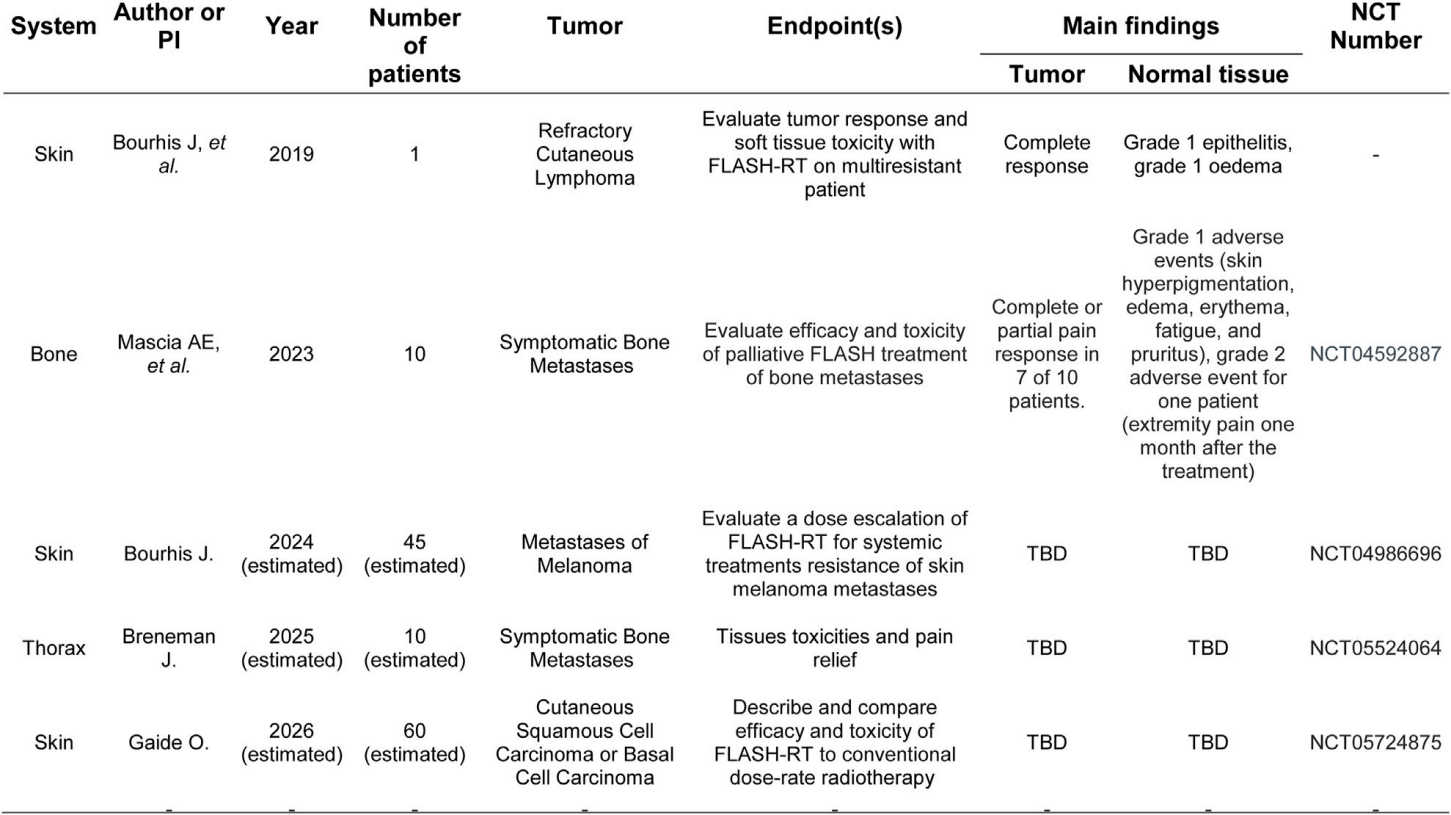
Clinical evidence of FLASH therapy effect.

The potential of FLASH radiotherapy is important. It can reduce normal tissue damage due to the limited time for normal cells to repair radiation-induced damage while increasing cancer cell killing. FLASH radiotherapy has also been associated with lower inflammation, and reduction in the exposure of healthy tissues to radiation may lower the risk of developing secondary cancers (91). See Figure 4 for the clinical evidence of FLASH effect Concerns include radiation-induced pulmonary fibrosis and treatment-related mortality (84). However, Favaudon et al. (92) demonstrated that mice exposed to ultrafast, single-pulse electron irradiation at 60 Gy/s did not develop pulmonary fibrosis, unlike mice exposed to conventional dose rates (0.03 Gy/s), which did develop fibrosis. This finding suggests a potential sparing effect at high dose rates (92).

### 6.2 History of the 4Rs/5Rs

Radiation therapy is based on the difference between a maximal TCP while minimizing the NTCP, which is defined as the therapeutic index. Various factors influence this index, which was originally described by Withers in 1975 as the “4 Rs” (93) and expanded by Steel in 1989 to the “5 Rs” (94).

#### 6.2.1 Repair of sublethal damage

One of the key researchers in this area was Ropolo, who, in 2009 (95). He showed that an extended cell cycle with prolonged checkpoint phases in stem cells may contribute to radioresistance. This phenomenon is very important to balance effective tumor control with minimal normal tissue toxicity (82).

#### 6.2.2 Cell cycle redistribution

A historic milestone in the late 1960s was the discovery of the G2-M phase as a radiosensitive phase, which was demonstrated in a clinical trial involving patients with head and neck cancer (96). The G2-M phase of the cell cycle is more radiosensitive because cells in this phase are actively preparing for mitosis. During this time, cells are more sensitive to radiation because of the consequences of errors in DNA repair, activation of the G2-M checkpoint, and the formation of mitotic spindles, all of which can lead to cell death (97).

Theoretical strategies, including the advent of fractionated radiotherapy in the 1970s (98), aimed to increase radiosensitivity by synchronizing cells in the G2‒M phase.

#### 6.2.3 Cellular repopulation

The activation of several pathways may increase the transcription of antiapoptotic and proliferative genes, contributing to radioresistance. Epidermal growth factor receptor (EGFR), a transmembrane protein discovered in 1962 (99), is crucial for driving cell proliferation and survival. Ionizing radiation induces the upregulation of EGFR expression as a protective mechanism, leading to increased cellular repopulation (100). The therapeutic targeting of EGFR was initiated in 1988. Mendelsohn et al. (101) showed that blocking EGFR activity with monoclonal antibodies could inhibit tumor growth. A study by Bonner et al. in 2006 demonstrated that the use of cetuximab, an EGFR inhibitor, in combination with radiation therapy significantly enhances local control and survival in patients with locally advanced head and neck cancer (102). FDA approval for cetuximab was given in 2004 (103).

#### 6.2.4 Tissue reoxygenation

Hypoxic cells exhibit 2- to 3-fold greater resistance to radiation. Since the pioneering work of Schwarz in 1909 (104), Holthusen in 1921, and Thomlinson and Gray in 1955 (105), oxygen has been recognized as a potent radiosensitizer. Oxygen enhances the effects of ionizing radiation by increasing free radical production through water radiolysis. This variability in tumor oxygenation levels contributes to the heterogeneous radiosensitivity of different cells (84). Monte Westerfield demonstrated in Zebrafish in 1995 that cell regeneration is enhanced through angiogenesis (53). Many efforts have been made to improve tissue oxygenation to improve the response to radiotherapy, such as the suppression of hypoxia-inducible factor 1α (HIF-1α) expression. Other strategies to mitigate tumor hypoxia and enhance radiotherapy efficacy include the inhibition of the recruitment of bone marrow-derived cells involved in vasculogenesis (55) and the suppression of angiogenesis through the blockade of vascular endothelial growth factor (VEGF). Bevacizumab, an inhibitor of VEGF receptor tyrosine kinase, was approved by the FDA in February 2004 (106).

#### 6.2.5 Intrinsic radiosensitivity

Divergent cellular responses related to radiosensitivity were first addressed by Hall et al. in 1972, who reported that the tissue response to radiation depends on cellular radiosensitivity and the dose/cell cycle, thereby incorporating the other 4 Rs (107). The concept of SF2 was introduced, namely, the fraction of cells surviving exposure to a single 2 Gy dose. The concept of SF2 is very important: in helps predict the response of tumor cells to radiation therapy. By measuring SF2, clinicians can tailor the treatment to individual patients. Cancers with low SF2 values are more radiosensitive, therefore respond better to radiation. Further exploration of these mechanisms could enhance personalized approaches in radiotherapy practice (108).

Recently there has been a sixth “R,” reactivation. Radiotherapy has long been considered to be immunosuppressive. However, it can trigger a systemic antitumor immune response following irradiation-induced immunogenic cell death. This is especially important with the advent of immunotherapy combined with radiation (109).

## 7 Treatments with high LET

Owing to its high LET and dense ionization along its path, it causes complex and clustered DNA damage. This supposedly causes a stronger reaction of the immune system against cancer than beta or gamma sources, or X-rays and electrons. The discovery of the Bragg Peak for protons by Robert Wilson in 1952 opened further possibilities for radiotherapy (110). Protons have been in use since the 1960s. In the 1970s, significant advances were made in other high-LET modalities (111). Heavy ion radiotherapy, including carbon ion therapy, has been used in Asia and Europe (112). The 2000s brought about further significant improvements to these technologies. Many centers worldwide have opened up proton treatments. Commercially available alpha irradiation is another new player that has opened new doors for radiation research. Owing to its high LET and dense ionization along its path, it causes complex and clustered DNA damage. This supposedly causes a stronger reaction of the immune system against cancer than beta or gamma sources, or X-rays and electrons (38). In 2020, the first clinical results of novel diffuse alpha-emitting radiation therapy (Alpha DaRT) using radioactive seeds implanted into tissues were published (113). One expects better insight into the interaction between irradiation and the immune system using this new high-LET technique (114).

### 7.1 The importance of women in radiobiology

One must underline the importance of women’s contribution to radiation biology. Marie Curie, who won two Nobel prizes opened the door for women in science in general. As mentioned above, the other important women were Rosalind Franklin and the south-African biologist Tikvah Alper, who later worked in London. In the early 1950s, she suggested that membranes were important targets of radiation. Another important female radiobiologist was Juliana Denekamp in England, who made major contributions to the design of radiotherapy schedules such as continuous hyperfractionated accelerated radiotherapy (CHART) and accelerated radiotherapy with carbogen and nicotinamide (ARCON) (115).

## 8 In conclusion

A comprehensive review of radiobiology over the past 120 years has revealed substantial progress in understanding the effects of radiation on both cancerous and healthy tissues. Despite these advances, several clinical observations remain poorly understood. Current theories do not fully explain all the clinical findings. Continued research is essential to elucidate these complex mechanisms and improve therapeutic outcomes. Significant progress can be expected from a combination of advances in molecular pathology and clinical radiobiology, as well as progress in radiation therapy planning and delivery technology (116).
